# Suicide in adolescence

**DOI:** 10.1192/j.eurpsy.2021.1675

**Published:** 2021-08-13

**Authors:** A. Zartaloudi, E. Kalligeri

**Affiliations:** 1 Nursing, University of West Attica, Athens, Greece; 2 Psychiatric Department, Sismanoglio General Hospital, Athens, Greece

**Keywords:** Suicidal ideation, adolescence, Suicide, self-harm

## Abstract

**Introduction:**

Suicide is one of the most common causes of death among young people worldwide. Adolescence is an important developmental period of life due to the increased risk of suicide and the prevalence of psychiatric disorders.

**Objectives:**

To explore the suicidal ideation, intentions and risk factors of adolescents.

**Methods:**

A clinical case study presentation will be performed.

**Results:**

An adolescent female, aged of 16 years old, was admitted to the Department of Psychiatry for Children and Adolescents of a General Hospital, diagnosed with behavioral and emotional disorder and active suicidal ideation on ground of sexual abuse. During her hospitalization, she exhibited self-destructive behaviour by swallowing objects or causing extensive skin scarring as well as serious suicide attempts by hanging. Her emotional and behavioral status was unstable and unpredictable. The adolescent had repeatedly expressed her will to escape from an unbearable life.

**Conclusions:**

The results of the presentation of our clinical case could contribute to the improvement of awareness regarding suicidal behavior in adolescence, which might have a significant effect on the prevention and treatment of this potentially lethal condition.

**Disclosure:**

No significant relationships.

